# Arthroscopic Deltoid Ligament Repair as a Potential Alternative Treatment for Ankle Deltoid Ligament Injury

**DOI:** 10.3390/jcm14051662

**Published:** 2025-02-28

**Authors:** Sung Hwan Kim, Sang Heon Lee, Joo Young Cha, Seung Won Choi, Young Koo Lee

**Affiliations:** Department of Orthopaedic Surgery, Soonchunhyang University Hospital Bucheon, 170 Jomaru-ro, Wonmi-gu, Bucheon-si 14584, Gyeonggi-do, Republic of Korea; shk9528@naver.com (S.H.K.); worldking70@naver.com (S.H.L.); cacarito@hanmail.net (J.Y.C.); tmddnjs4836@naver.com (S.W.C.)

**Keywords:** deltoid ligament injury, arthroscopic deltoid repair, open deltoid repair

## Abstract

**Background:** Arthroscopic deltoid ligament (DL) repair is a recently introduced technique, with few studies currently comparing the outcomes of open and arthroscopic deltoid repairs. This study compares the clinical and radiologic outcomes of patients who underwent either open or arthroscopic DL repair. **Methods:** Forty-one patients underwent surgical repair for a ruptured DL by a single surgeon at the study site hospital between 2013 and 2022. Clinical outcomes were assessed using the Foot and Ankle Outcome Score (FAOS), the American Orthopedic Foot and Ankle Society (AOFAS) Ankle–Hindfoot Scale, and a visual analog scale (VAS). Radiologic outcomes were evaluated through anterior talar translation and talar tilt tests, with stress radiography conducted at 6 months and 1 year post-surgery. **Results:** No significant differences in sex ratio, age, or direction of injury were observed between the groups. Additionally, there were no significant differences in clinical and radiologic outcomes between the groups. However, both clinical and radiologic outcomes showed significant improvement after surgery compared to preoperative conditions in both groups. **Conclusions:** Considering the benefits of arthroscopic surgery, arthroscopic deltoid repair can be regarded as a suitable option for treating DL injuries.

## 1. Introduction

The ankle is a common site for sports injuries [1]. According to the ankle fracture classification by Lauge-Hansen, injuries to the deltoid ligament (DL) or fractures of the medial malleolus occur as the injury progresses around the ankle in a circular pattern [2]. The three primary mechanisms of injury to the DL are foot pronation-abduction, pronation-external rotation, and supination-external rotation [1,2,3]. Among these, injuries resulting from supination-external rotation are the most prevalent [1]. The DL is a robust, multi-banded complex comprising both superficial and deep components that stabilize the ankle. The superficial layer of the DL consists of the tibionavicular, tibiospring, and tibiocalcaneal ligaments, all of which traverse the ankle and subtalar joints [4,5]. A recent anatomical study examined the composition of the DL and revealed that the tibio-calcaneonavicular spring ligament is part of the superficial deltoid [6,7]. The anterior and posterior tibiotalar ligaments are part of the deep deltoid layer and exclusively cross the ankle joint [4,5]. The superficial deltoid resists hindfoot eversion, while the deep deltoid restrains external rotation of the talus [8]. All segments of the DL collaborate to provide static support to the ankle throughout its movement. The deep DL has been identified as the main contributor to ankle stability against valgus stress, while the superficial DL has a minimal impact on overall stability [5,9,10]. However, a recent investigation suggested that repairing the anterior DL (including the talonavicular and tibiospring ligaments) effectively restricts lateral talar movement after surgery. This finding indicates that the anterior DL may contribute more significantly to medial stability than was previously understood [5].

Ruptures of the DL often accompany ankle fractures, with up to 40% of ankle fractures showing concurrent DL injuries upon arthroscopic examination [11]. Recent research has underscored the value of ankle arthroscopy for diagnosing and treating intra-articular joint damage during the surgical reduction and fixation of fractures [12]. This damage may include loose fragments, osteochondral defects, impingement lesions, and instances of syndesmosis malreduction or instability. Arthroscopy provides a direct view of the DL’s condition and the extent of any tears. Instability becomes apparent during arthroscopy through increased anterior-to-posterior motion under stress or when a rotational stress test is conducted on an externally rotated and everted ankle, resulting in a widened medial gap [11]. In addition, arthroscopic surgery is much more advantageous from a cosmetic perspective because it requires fewer incisions. We aimed to evaluate the feasibility of mending the DL using an arthroscopic approach [11,13]. The objective of arthroscopic DL repair is to expedite postoperative recovery, facilitate earlier resumption of full functionality, and prevent the development of persistent medial and rotational ankle instability [5]. We propose that repairing the superficial DL can address instability resulting from ruptures of both the deep and superficial DL. Moreover, medial instability caused by DL rupture can be easily detected and corrected arthroscopically. There are also claims that DL repair using arthroscopy may be less stable than open repair [6,10]. However, when the authors observed clinically, they found that the difference between the two techniques was not significant and conducted this study to theorize this.

Between 2013 and 2022, we assessed the clinical outcomes of patients who underwent open and arthroscopic deltoid repair surgery in an outpatient setting, and both treatment approaches yielded excellent results. Therefore, we hypothesized that arthroscopic deltoid repair would produce favorable outcomes, leading us to design a comparative study.

## 2. Methods

### 2.1. Patient Selection

The study was carried out in accordance with the Declaration of Helsinki and received approval from the Institutional Review Board and Human Research Ethics Committee of the study site hospital (IRB No. 2023-07-002, 11 July 2023). Patient consent was waived in consideration of the retrospective nature of the study.

We retrospectively enrolled all patients who received surgery for a ruptured DL by a single surgeon at the study site hospital between 2013 and 2021. The study involved skeletally mature patients with ruptured DL, available postoperative plain and stress radiographic images, and a follow-up duration exceeding 1 year. We excluded patients with open fractures, polytrauma, previous ankle fractures, neurological impairments in the lower limbs, and ankle inflammatory diseases, as these conditions can affect patient outcomes.

Plain anteroposterior, lateral, and stress radiographic views were obtained to evaluate the medial stability of the ankle by measuring the medial clear space (MCS). A DL injury was suspected if the MCS was greater than 4 mm or at least 1 mm greater than the superior tibiotalar clear space on plain radiography without a stress test [14]. Magnetic resonance imaging was performed on all patients to evaluate the extent of ligament injury and identify any associated intra-articular pathologies. These images were also used for preoperative planning. Open surgery was required to internally fix concomitant fractures in cases of DL ligament injury due to trauma. Since an incision was already made for the open reduction and fixation of the fracture, an open deltoid repair was performed during the surgery. For patients without additional injuries, a minimally invasive arthroscopic deltoid repair was conducted without the need for extra incisions. Intraoperative plain and stress radiographs were obtained using a C-arm image intensifier after surgical repair of the DL to confirm the medial stability of the ankle.

### 2.2. Open Deltoid Repair Technique

**Figure 1:**
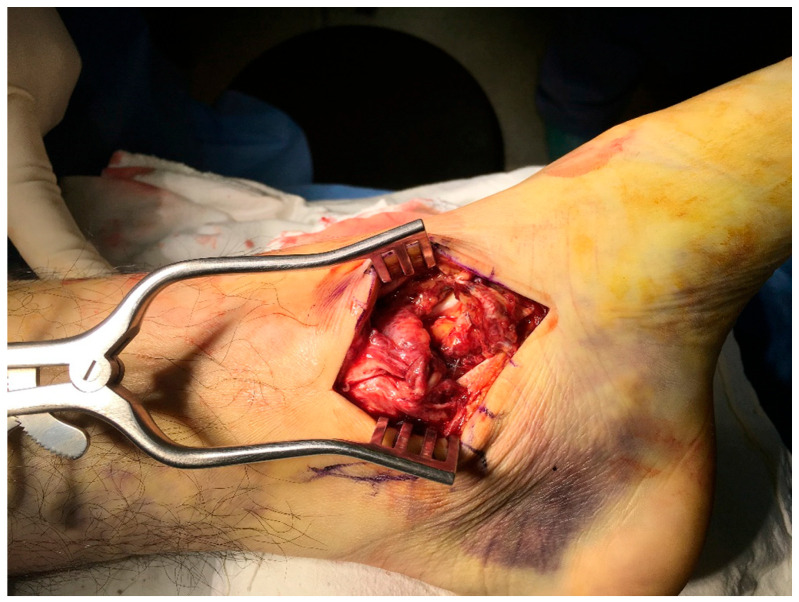
Photograph of a completely ruptured deltoid ligament; a curvilinear 5 cm incision was made for the repair.

A curvilinear incision measuring 5 cm was made just below the medial malleolus. After retracting the tibialis posterior tendon plantarly, the deep fascia was incised, exposing the superficial and deep fibers of the DL. Typically, one or two suture anchors (TruShot with Y-Knot; CONMED, Largo, FL, USA) were inserted into the anterior and/or posterior colliculi of the medial malleolus. The suture anchors were placed beneath the bony surface, and wire sutures were utilized to create a full-thickness, horizontal mattress suture pattern in the DL complex for direct repair. After positioning the sutures from the anchors and applying the appropriate tension, the surrounding soft tissues were imbricated to provide additional support. The ankle was slightly inverted during the repair to reduce tension on the deltoid complex. Fluoroscopy was almost always employed to ensure correct positioning of the anchors and the talus within the mortise. Manual stress testing was frequently performed to confirm medial stability (Figure 1).

### 2.3. Arthroscopic Deltoid Repair Technique

**Figure 2:**
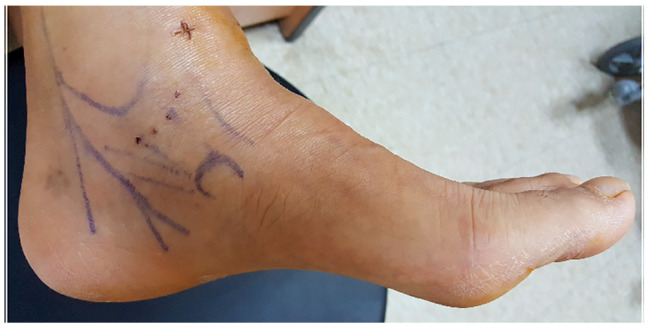
Photograph of the foot after arthroscopic deltoid ligament repair. All suture portals should be positioned within the “the safe zone” between the saphenous and tibial nerves.

**Figure 3:**
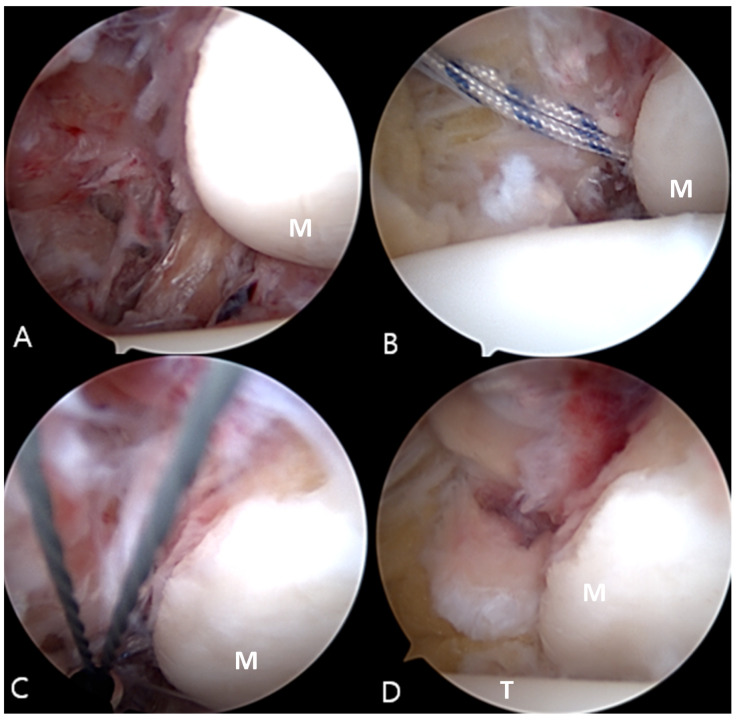
Arthroscopic photographs of deltoid ligament repair: (**A**) predrilling to insert the absorbable TruShot (CONMED) anchor; (**B**,**C**) all sutures were pulled through the distal portion of the anterior deltoid ligament; (**D**) repaired deltoid ligament. T, talus; M, medial malleolus.

The patient was positioned supine on the operating table under general or spinal anesthesia, and the tourniquet was inflated. The deep DL was evaluated using the anterolateral and anteromedial portals. The synovial tissue and periosteum were excised immediately distal to the anterior DL to facilitate anchor insertion, exposing the bleeding bony surface of the medial malleolus with a motorized burr. A drill hole was made perpendicular to the anterior surface of the anterior colliculus, and the anchor was inserted through the anteromedial portal. An absorbable anchor (TruShot with Y-Knot; CONMED) was equipped with two sutures. Each suture was threaded through the distal portion of the anterior DL, located between the tibialis anterior and posterior tendons, and positioned within “the safe zone” between the saphenous and tibial nerves (Figure 2). The knots were secured with the foot in an inverted and dorsiflexed position. Finally, the portals were sutured using standard technique (Figure 3).

### 2.4. Radiographic Evaluation

All patients had a follow-up period of over 1 year, during which serial clinical examinations and radiographs were conducted. Plain radiography was performed preoperatively, immediately after surgery, 6 months after surgery, and 1 year after surgery, while stress radiography was conducted only at 6 months and 1 year post-surgery. Stress radiographs were obtained with the foot positioned at up to 10° of plantarflexion. Anterior drawer test (ADT) measurements were taken from lateral radiographs with the leg held in slight internal rotation. During the acquisition of these stress radiographs, a load of 150 N was applied to the ankles in both the ADT and talar tilt planes, with patients instructed to relax their leg muscles completely. The MCS was assessed as the distance from the medial malleolar articular surface to the medial talar surface 1 cm below the talar dome. MCS measurements were performed by three different authors of the present study.

### 2.5. Clinical Outcomes

Functional outcomes were assessed using the Foot and Ankle Outcome Score (FAOS), the American Orthopedic Foot and Ankle Society (AOFAS) clinical rating system, and a visual analog scale (VAS). The FAOS consists of five domains: symptoms, pain, activities of daily living, sports, and recreation. Quality of life is evaluated in terms of functional limitations in daily activities and subjective symptoms among patients with foot and ankle disorders. The AOFAS includes four scales related to pain and function in the ankle–hindfoot, midfoot, hallux metatarsophalangeal–interphalangeal, and lesser metatarsophalangeal–interphalangeal regions. These scales require physicians to conduct alignment, gait, and motion examinations. The VAS was employed to assess subjective pain. All functional and clinical evaluations were conducted preoperatively and 6 months and 1 year post-surgery.

### 2.6. Statistical Methods

Continuous variables were assessed using the Shapiro–Wilk test, which indicated that the data were not normally distributed; therefore, nonparametric statistical tests were employed for comparisons. Continuous variables are described as medians with the 25th and 75th percentiles, while categorical variables are presented as frequencies and percentages. Variables were compared between independent groups using the Wilcoxon rank-sum test and Fisher’s exact test, as appropriate. Changes in each variable over time were evaluated using the Wilcoxon signed-rank test. All tests were two-tailed, and *p*-values < 0.05 were considered significant. Statistical analyses were conducted using Rex software (version 3.0.3; RexSoft Inc., Seoul, Republic of Korea). Intraclass correlation coefficients (ICCs) were calculated to estimate intra-rater reliability, with the degree of reliability deemed almost perfect according to the ICC scale proposed by Landis and Koch [15].

## 3. Results

Eighty-nine patients underwent surgical repair for a ruptured DL by a single surgeon at the study site hospital between 2013 and 2022. Twenty-one patients were excluded from the study due to the following reasons: open fractures (*n* = 10), polytrauma (*n* = 6), previous ankle fractures (*n* = 2), neurological impairments of the lower limbs (*n* = 2), or inflammatory diseases of the ankle (*n* = 1). Among the remaining patients, 68 were followed up in outpatient clinics for at least one year and had available clinical and radiological outcome data; 27 were excluded due to missing postoperative radiographs or survey data. Ultimately, 41 patients (19 women, 46%) were included in the analysis: 22 underwent arthroscopic deltoid repair, while 19 underwent open deltoid repair. The median ages of patients in the arthroscopic and open repair groups were 32 and 40 years, respectively. The right ankle joint was injured in 14 (34%) patients.

Table 1 presents the demographic characteristics of the patients in the study groups. No significant differences were found in sex ratio, age, or direction of injury between the groups.

Table 2 presents a comparison of the clinical outcomes at 6 months and 1 year postoperatively between the two groups. Both groups had a median AOFAS score of 90 after 1 year. The median FAOS in the arthroscopic and open repair groups after 1 year was 59 and 56, respectively. The median VAS scores for the arthroscopic and open repair groups were 2 and 1, respectively. These findings indicate no significant differences between the groups.

**Figure 4:**
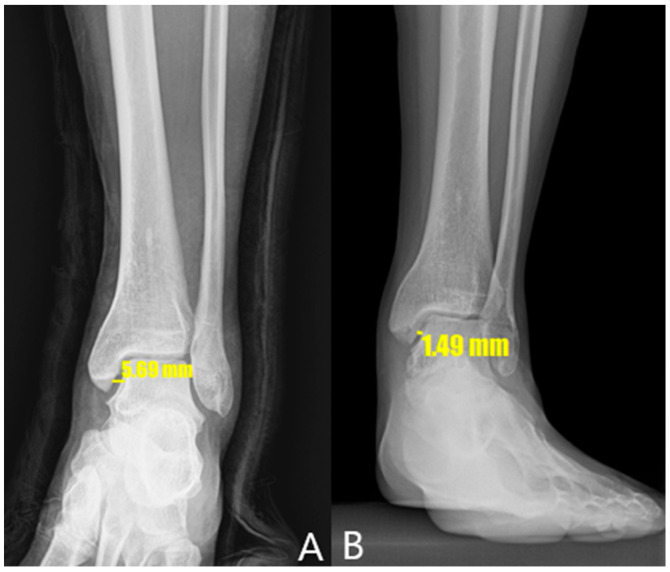
X-ray of one patient who participated in this study. (**A**) MCS value before surgery. (**B**) MCS value has improved 1 year after surgery.

Table 3 compares the radiological outcomes between the two groups. The preoperative MCS values in the arthroscopic and open repair groups were 3.1 mm and 6.56 mm, respectively. The immediate postoperative MCS values in the arthroscopic and open repair groups were 2.2 mm and 2.53 mm, respectively. The median MCS values after 1 year in the arthroscopic and open repair groups were 2.21 mm and 2.24 mm, respectively. The median MCS values measured by stress radiography at 1 year in the arthroscopic and open repair groups were 2.62 mm and 3.07 mm, respectively. These findings indicate significant differences in the preoperative, immediate postoperative, and 1-year postoperative MCS values between the groups, while no significant differences were detected in the postoperative MCS values at 6 months or 1 year. Additionally, the MCS values measured by stress radiography at both 6 months and 1 year significantly differed between the two groups. However, the improvement in MCS values before and after surgery in both groups means that the radiological results after surgery were better than before surgery for both methods. In Figure 4, an X-ray of one of the patients who participated in this study shows that MCS value had improved after surgery.

## 4. Discussion

The results of this study showed no significant difference between arthroscopic deltoid repair and open deltoid repair. Additionally, clinical outcomes improved significantly after surgery compared to preoperative conditions in both groups. In particular, MCS at 6 months and 1 year after surgery was shown to be more stable in the arthroscopic group.

The question of whether a ruptured DL associated with a malleolar fracture should undergo repair remains controversial [16]. However, a recent trend suggests that such injuries could lead to enduring pain or pronation deformities [5,17,18]. The fundamental principles of managing ankle fractures involve restoring anatomical alignment and joint congruity, ensuring stability, and minimizing long-term complications. The medial column, which comprises the medial malleolus and DL, is more critical for ankle stability than the lateral counterpart. The DL acts as a medial stabilizer that anchors the talus and guides its normal range of physiological motion [19]. Disruption of the DL complex can cause lateral migration or tilting of the talus within the mortise. Some studies have demonstrated that even minor deviations in anatomical alignment lead to substantial reductions in joint contact area [20]. As the overall contact area diminishes, stress per unit area increases, theoretically amplifying strain on specific regions of the ankle joint during normal loading [16]. Consequently, medial instability arising from a DL rupture can result in adverse outcomes for concurrent ankle fractures and complications, such as osteoarthritis [16,20].

In 1972, Weber proposed that surgical intervention for ankle injuries should commence with an assessment of the medial aspect of the ankle, followed by DL repair. He suggested that the anatomical restoration of the lateral ankle might be unfeasible if the DL was interposed [21]. In contrast, both Heim and De Souza et al. contended that exploring the medial ankle was unnecessary when reconstructing the ankle mortise [22,23]. Several case series have reported DL repair in the context of ankle fractures accompanied by deltoid injuries [16,24]. Their findings indicate favorable outcomes without complications.

Recent investigations have highlighted the significant role of early DL repair [12,16]. Several researchers have emphasized that non-anatomical healing of a DL complex injury can adversely affect clinical and functional outcomes in both short- and long-term evaluations [12,25]. Consequently, patients may experience persistent medial ankle discomfort following bimalleolar equivalent injuries, potentially arising from medial instability due to non-operative management of the DL [4]. Moreover, neglecting to repair the DL could result in ankle joint misalignment, potentially leading to osteoarthritis. Notably, a recent study by Woo et al. indicated that immediate repair of a deltoid injury yields a significantly reduced final MCS during subsequent follow-up assessments [12].

The MCS refers to the anatomical gap between the outer side of the medial malleolus and the inner side of the talus [26]. It is frequently used in foot and ankle surgery to assess the competence of the DL. A widened MCS indicates DL incompetence, necessitating surgical intervention to prevent lateral talar shift. MCS is commonly evaluated through radiographic views of the ankle, and various methods for measuring the MCS have been described in the literature. As mentioned earlier, in our study, the MCS was measured as the distance from the medial malleolar articular surface to the medial talar surface 1 cm below the talar dome. There are multiple definitions of medial instability in the literature, including an MCS ≥ 4 mm and 1 mm greater than the superior tibiotalar space, an MCS ≥ 5 mm, and an increase in MCS of 2 mm from its baseline value [13,27]. Additionally, for ankles with an MCS between 4 and 5 mm, instability should be considered only if there is a lateral shift greater than 2 mm on stress examination [28]. Possible variations in MCS measurements may arise due to factors such as sex, height, foot position, and the type of radiography used. Tiago et al. noted that plantar flexion significantly impacts the MCS, leading to an increase in its measurement [29].

Notably, Kim et al. and Vega et al. recently introduced methods for the arthroscopic repair of the DL [5,30]. The most precise assessment of medial instability is achieved through arthroscopic visualization, allowing for the repair of the DL while simultaneously addressing intra-articular abnormalities using a minimally invasive approach. Moreover, this surgical technique offers cosmetic benefits due to its minimally invasive nature, resulting in smaller scars.

There are several limitations of our study. First, our study included a relatively small number of patients, which may have affected the statistical significance of the results. Due to this limited sample size, we could not distinguish between patients who had repairs due to fractures and those who had repairs due to ruptures. As there are differences in the mechanism of injury and surgical methods between the arthroscopic and open repair groups, a more detailed comparison of these two groups is necessary for accurate postoperative evaluation. Another limitation is the variation in preoperative injury severity between the two groups, as assessed by preoperative MCS values. The open surgery group tended to exhibit higher severity compared to the arthroscopic group. This discrepancy in initial injury severity could affect the accuracy of the comparisons and introduce bias, potentially confounding the evaluation of surgical outcomes. Consequently, this variation may impact the reliability of the comparative analysis between the two techniques. Furthermore, our ability to predict complications and outcomes of surgical treatments, such as osteoarthritis, instability, and medial gutter pain, may be limited since these issues could manifest beyond the 1-year follow-up period for clinical and radiological assessments. Therefore, long-term prospective studies involving a larger patient population are necessary.

## 5. Conclusions

Both open and arthroscopic repair techniques for DL injuries yielded clinically and radiologically satisfactory outcomes, with no significant differences between the two groups. Considering the advantages associated with arthroscopic surgery, arthroscopic deltoid repair should be regarded as a favorable surgical approach for DL injuries.

## Figures and Tables

**Table 1:** Demographic characteristics of the patients in the arthroscopic and open repair groups.

**Variable**	**Arthroscopic** **(*N* = 22)**	**Open** **(*N* = 19)**	***p*-Value**
Age	32.5 (24.25–48.75)	40.5 (31.53.5)	0.082
Sex			0.613
Female	11 (50)	11 (57.89)	
Male	11 (50)	8 (42.11)	
Direction			0.735
Left	15 (68.18)	12 (63.16)	
Right	7 (31.82)	7 (36.84)	

Data are presented as *N* (%).

**Table 2:** Clinical outcomes in the arthroscopic and open repair groups.

**Variable**	**Arthroscopic** **(*N* = 22)**	**Open** **(*N* = 19)**	***p*-Value**
POM 6 AOFAS	88 (72.25–92.25)	86 (80.5–91.5)	0.6365
POM 6 VAS	2.5 (2–4)	2 (1–3)	0.2459
POM 6 FAOS	65.5 (53–85.75)	65 (55.5–76)	0.7536
POY 1 AOFAS	90 (80.75–100)	90 (87.5–99)	0.9363
POY 1 VAS	2 (1–2)	1 (1–2.5)	0.8192
POY 1 FAOS	59 (45–80.25)	56 (52–70)	0.9374

AOFAS American Orthopedic Foot and Ankle Society; FAOS Foot and Ankle Outcome Score; POM postoperative month; POY postoperative year; VAS visual analog scale.

**Table 3:** Radiological outcomes in the arthroscopic and open repair groups.

**Variable**	**Arthroscopic** **(*N* = 22)**	**Open** **(*N* = 19)**	***p*-Value**
Preop MSC	3.1 (2.48–3.64)	6.56 (3.37–9.07)	0.0047
Postop MCS	2.2 (2.69–2.34)	2.53 (1.99–3.15)	0.0228
POM 6 MCS	2.13 (1.83–2.56)	2.21 (2.04–2.79)	0.1823
POM 6 stress	2.67 (2.23–3.06)	3.27 (2.5–3.97)	0.0427
POY 1 MCS	2.21 (1.95–2.45)	2.24 (1.79–2.67)	0.9479
POY 1 stress	2.62 (2.23–2.92)	3.07 (2.75–3.56)	0.018

POM postoperative month; postop MCS immediate postoperative medial clear space; POY postoperative year; preop MCS preoperative medial clear space; stress medial clear space measured on valgus stress radiographs.

## Data Availability

Data sharing is not applicable to this article because no datasets were made or analyzed during this study.
